# The Presence of a ‘Sentinel’ Vessel as an Anatomical Reference during Hamstring Tendon Harvesting—A Prospective Study

**DOI:** 10.3390/jcm12165426

**Published:** 2023-08-21

**Authors:** Radu Prejbeanu, Mihail Lazar Mioc, Silviu Jebelean, Andrei Balanescu, Andrei-Marian Feier, Tudor Sorin Pop, Octav Russu

**Affiliations:** 1 Department of Orthopedics, “Victor Babes” University of Medicine and Pharmacy Timisoara, 300041 Timisoara, Romaniamihail.mioc@umft.ro (M.L.M.); 2 Department of Orthopedics and Traumatology, Premiere Hospital Timisoara, 300643 Timisoara, Romania; dr.jebelean@yahoo.com (S.J.); andreidan.balanescu@gmail.com (A.B.); 3 Department of Orthopaedics and Traumatology, “George Emil Palade” University of Medicine, Pharmacy, Science, and Technology of Targu Mures, 540142 Targu Mures, Romaniaoctav.russu@umfst.ro (O.R.)

**Keywords:** pes anserinus, ACL anatomic landmarks, hamstring harvesting, ACL reconstruction, ACL graft harvesting

## Abstract

Background: The identification of the branch of the inferior medial genicular artery (bIMGA) in anterior cruciate ligament reconstructions (ACLRs) has previously been considered a landmark by some surgeons, but its consistency remains debated. The aim of this investigation was to evaluate the variability in the appearance and location of bIMGA and to assess its validity as a reliable landmark during hamstring tendon harvesting procedures. Methods: This prospective, single-center study comprised 213 patients who underwent ACLR over a period of two years. The surgical procedures were conducted by the same surgical team, maintaining uniformity in the approach. The study sought correlations between patient demographics, level of activity, and the potential for successful identification of the bIMGA. Results: A statistically significant association between patient activity levels and successful identification of the bIMGA (*p* = 0.035) was observed. No significant correlations were found concerning patient demographic characteristics. bIMGA demonstrated a substantial degree of anatomical variability, rendering its consistent identification in the surgical field challenging. Conclusions: Given the observed variability and the associated difficulty in its identification, the use of the bIMGA as a dependable anatomical reference during ACL graft harvesting is not recommended. This study confirms the inconsistency of bIMGA as a traditional landmark, underscoring the need for research aimed at identifying more consistent and reliable anatomical references to enhance the precision of surgical interventions in ACLR.

## 1. Introduction

The anterior cruciate ligament (ACL) is essential for knee stability and function. Injuries to the ACL can impair mobility and necessitate surgical intervention, making ACL reconstruction (ACLR) a common and vital orthopedic procedure, particularly in the context of sports-related injuries [1,2].

Over the years, surgical techniques and graft choices have evolved considerably, offering a wider array of options to orthopedic surgeons and patients alike [3,4]. A successful ACLR heavily relies on optimal graft integration, which can be influenced by several factors, including the choice of autograft and other inherent biological considerations [5]. Among the multitude of available graft sources, autografting using the patient’s own hamstring tendons (HT) remains a widely favored choice due to its proven track record of positive clinical outcomes and lower graft rejection risks [5,6]. Due to its potential to influence the overall surgical outcome and the patient’s post-operative recovery, graft harvesting is a nuanced and intricate step in the ACLR procedure [5]. It calls for a high level of surgical skill and precision, combined with a comprehensive understanding of knee anatomy [6]. In the nuanced and complex procedure of graft harvesting for ACLR, specific anatomical landmarks serve as essential guides. The identification of the medial border of the tibia, the tibial tubercle, and the sartorial fascia (after dissecting superficial tissues) provides surgeons with orientation points. These landmarks have been instrumental in streamlining the predictability of the procedure, thereby minimizing associated complications [7,8].

A comprehensive understanding of the pes anserinus anatomy is paramount in the ACLR graft harvesting procedure. Significant insights into the vascularity and neuroreceptors of this notable landmark were provided in a comprehensive study conducted two decades ago [7]. Any inaccuracy in delineating tendons necessary for grafting can lead to serious complications, including damage to the surrounding soft tissues or neurologic pathology via injury to the infrapatellar branch of the saphenous nerve [8,9]. Furthermore, the necessity to create a clear demarcation between the sartorius muscle tendon and the tendons of the gracilis and semitendinosus muscles cannot be overstated, as it is integral to the success of the graft harvest and subsequent reconstruction [10].

**Figure 1:**
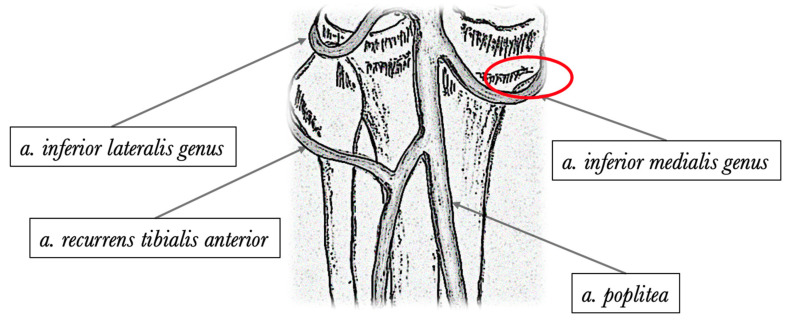
Posterior anatomical illustration depicting the primary genicular arteries. An area delineated by a red oval indicates the typical branching point of the inferior medial genicular artery.

Recently, a novel vascular anatomical reference, frequently named the “sentinel vessel” or the inferior medial geniculate artery branch (bIMGA), has been brought to the forefront of ACLR surgical planning, with studies suggesting its potential to enhance procedural predictability and decrease harvest-related complications [11,12,13]. The inferior genicular arteries, consisting of the lateral and medial inferior genicular arteries and their branches are critical vascular structures that arise from the popliteal artery (Figure 1).

They are primarily responsible for supplying blood to the knee joint, complementing the contributions from their superior genicular counterparts [13]. However, these studies also propose that this vascular structure may not be universally present, posing a potential challenge to its widespread application. This limitation highlights the need for careful consideration and potential alternatives when planning the surgical approach, recognizing that its absence might impact the velocity and outcome of the procedure.

The main objective of the present study is to investigate the anatomical variability of this vascular structure in the context of ACLRs. Insight is sought on its incidence and implications for surgical planning and procedure standardization. A secondary aim is to augment the state-of-the-art and assist in the refinement of ACLR surgical techniques.

## 2. Materials and Methods

The conduct of this study was in full compliance with ethical standards, having received formal approval (No. 2023334) from the local ethical review board at Regina Maria Hospital’s ethics committee, located in Timisoara. This process included a thorough review of the research protocols to ensure the safeguarding of participant rights, adherence to the principles of informed consent, and compliance with relevant regulatory standards. Furthermore, all research procedures strictly adhered to the principles of the Declaration of Helsinki.

### 2.1. Study Design

A prospective study was conducted involving 213 consecutive patients who underwent ACLR with an HT autograft. The study participants had experienced an isolated knee trauma leading to an ACL tear, which was confirmed via magnetic resonance imaging (MRI) and clinical examination. The surgical procedures were conducted by the same surgical team, led by a senior orthopedic surgeon. Assisting him were two junior orthopedic surgeons. The combined expertise of this team ensured uniformity and precision in the approach over a period of approximately 24 months.

### 2.2. Inclusion and Exclusion Criteria

The study included patients who had experienced an isolated knee trauma resulting in an ACL tear, confirmed by MRI and clinical examination (Lachman and anterior drawer tests). Both male and female patients aged between 18 and 65 years were considered for inclusion. Additionally, the patients had to range from very low to very high on a physical activity scale from 1 to 5. Patients were excluded from the study if they had multiple ligament injuries in the knee or had undergone previous surgeries on the affected knee. We also excluded those with systemic diseases or conditions that could interfere with the surgery or post-operative recovery, such as diabetes, rheumatoid arthritis, or any form of immunodeficiency. Patients who were unable or unwilling to follow post-operative care instructions or to attend follow-up appointments were not considered for the study.

### 2.3. Surgical Procedure

Surgical procedures were performed with patients in a supine position, with the knee flexed and placed in a leg holder, under regional anesthesia. A tourniquet was uniformly used, inflated at 275 mmHg following vascular drainage of the leg and prior to skin incision. Prophylactic antibiotic therapy and tranexamic acid were administered during surgery.

**Figure 2:**
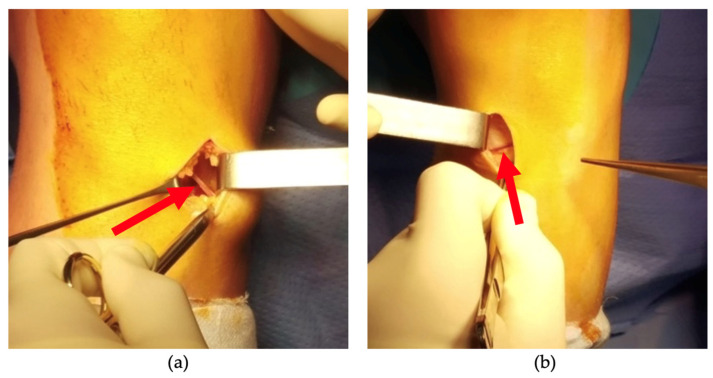
Intraoperative image taken of knees where bIMGA is present (**a**,**b**). The direction and orientation of the vessel can be observed (red arrow).

**Figure 3:**
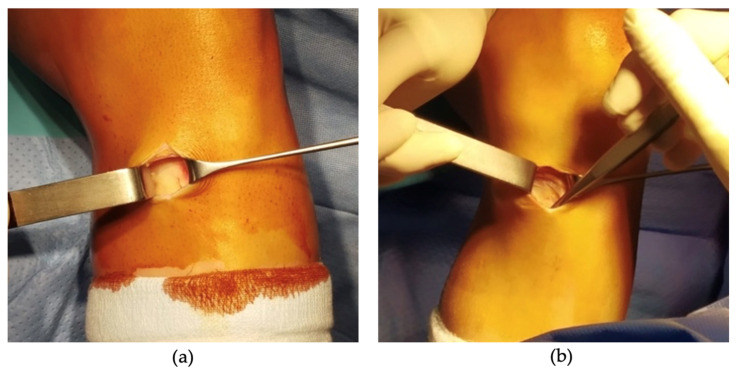
Intraoperative images taken of knees with the absence of bIMGA (**a**,**b**).

A small incision was made over the pes anserinus, where the tendons insert on the medial side of the tibia, just below the knee joint line. Identification of the pes anserinus was achieved by palpating 1.5 cm medial and below the lower margin of the tibial tubercle. The skin incision typically ranged from 2 to 3 cm in length, positioned slightly oblique from medial and proximal to lateral and distal. Subsequent to meticulous dissection of the subcutaneous tissues, a Langenbeck retractor was placed medially and the presence or absence of the ‘sentinel vessel’ was noted and documented (Figure 2 and Figure 3).

The HT harvesting continued with a horizontal incision of the fascia and identification of the gracilis and semitendinosus tendons at their insertion points on the pes anserinus. A closed tendon stripper was utilized to strip the grafts proximally, and the HT preparation proceeded with muscle tissue stripping and quadruple stitching of the graft. The process culminated with a standard, single-bundle graft ACL reconstruction.

### 2.4. Statistical Analysis

Data processing was performed with the SPSS^®^ 26 Suite (IBM© Corporation, Armonk, NY, USA, 2019). Descriptive analyses were conducted to determine frequencies and correlations. Both the Student’s *t*-test and the chi-square tests were used, and statistical significance was established for each variable. The *t*-test was selected as the statistical analysis method due to its efficacy in comparing means between two independent groups, which in this case were the patient groups. Before the implementation of the *t*-test, a Shapiro–Wilk test was conducted to ascertain the normality of the data distribution. Additionally, the homogeneity of variances was confirmed using Levene’s test, another assumption for the *t*-test, to ensure the reliability of the analysis outcomes. Sensitivity analyses were conducted to explore the robustness of the findings, providing added confidence in the results. All findings were presented with a confidence interval of 95%, reflecting the level of certainty in the statistical conclusions drawn from the study.

In conducting our analysis, we first segregated the dataset into two groups based on the gender of the patients: male and female. The variable of interest was the proportion of patients presenting with bIMGA in each group. For each gender, we calculated the mean proportion of patients with bIMGA across all age groups. We then employed the independent samples *t*-test to compare these mean proportions between males and females. This test was chosen because it allows the comparison of means between two independent groups and our data met the assumptions of normality and equal variances.

## 3. Results

In the study population, 135 individuals (63.4%) were male, exhibiting a mean age of 30.2 years, with an age range of 15–46 years, and were predominantly involved in active sports or physical occupations. Patient characteristics and demographics, including details about their social determinants of health, are illustrated in Table 1.

Preoperative data were collected at the time of study enrollment by a study nurse and are meticulously presented in Table 2. Given the significant influence that age exerts on the vascular tonus, which is known to be a crucial factor in various physiological responses, we have chosen to present it more granularly, separately in a distinct table, along with activity level. This method of presentation allows for a more detailed examination of age-related variations and their potential implications on the study’s findings.

A diminished degree of consistency was encountered in the identification of the bIMGA within the patient cohort when utilizing the described surgical procedure. Patient subgroups were established based on the presence or absence of the specified blood vessel. The data associated with these groupings were systematically accumulated and are presented in Table 3. No statistically significant correlations could be discerned between patient characteristics and the presence or absence of the bIMGA.

The results of the t-test indicated that there was no statistically significant difference between the mean proportion of males and females presenting with bIMGA across all age groups (Table 4). As a result, our test did not identify a statistically significant difference between the two means, indicating that the gender of the patients did not have a significant effect on the proportion of bIMGA presentation across all age groups in our study. The results of the chi-square test indicated a possible association between the age group and the presence or absence of bIMGA. This correlation, however, did not attain statistical significance.

The chi-square test was used for data pertinent to specific levels of activity. The calculated *p*-value was 0.035, which was less than the significance threshold of 0.05. Both genders exhibit a statistically significant correlation between activity level and the presence or absence of bIMGA.

## 4. Discussion

The main discovery in this investigation is the correlation between the patient’s activity levels and the successful identification of the bIMGA. The observed association suggests that there may be underlying factors related to activity levels that coincide with the identification of the bIMGA It is essential to note that this finding does not imply a causal relationship between physical activity and the detectability of this vascular structure during ACL graft harvesting. The complexities associated with the identification and obtaining appropriate length tendons from the pes anserinus are well documented [14]. Precision is of the utmost importance during this phase, as it substantially influences the success rate and surgery time of the ACL reconstruction procedure [15]. In light of this, it is imperative to employ rigorous and accurate techniques for the identification of pes anserinus, a procedure intrinsically linked to the meticulous delineation of the precise muscle insertions, namely the gracilis and the semitendinosus [16]. Identification and differentiation are based on well-established anatomical landmarks, with the tibial tubercle serving as the primary reference point [17]. It facilitates the accurate identification of pes anserinus and subsequent isolation of the desired tendons by acting as a consistent and dependable guide.

The presence of tendon anatomical variations demands significant consideration. Numerous research studies have identified a variety of anomalies, including accessory fascial bands, supernumerary muscles, and additional tendon slippage, thereby posing difficulties in the precise procurement of tendons for grafting [18,19,20]. The inability to harvest adequately due to these variations may result in suboptimal autografts, which may necessitate a switch to an allograft or an instantaneous change in the autograft selection during the procedure. In addition to extending the duration of surgical procedures, such occurrences may also contribute to higher rates of complications, thereby adding to the complexity of the procedure. A retrospective study that used MRI to evaluate adolescents who have undergone ACL reconstruction revealed the severity of this issue [14]. The research discovered that approximately 3% of the adolescent research cohort had anomalous tendons. This highlights the need for comprehensive preoperative evaluations, a better understanding of anatomical variations, and a commitment to ongoing research in this area with the final aim to refine surgical strategies and optimize personalized patient outcomes.

Recent research by de Lima Lopes and colleagues [12] revealed the existence of a vascular arch originating from three primary arteries: the medial inferior geniculate artery, the lateral inferior geniculate artery, and the anterior recurrent tibial artery. This complex vascular network characterized by variable vessel diameters was observed in all study participants. Despite these results, the reliability of this vascular arch as an anatomical reference was still questionable. A crucial limitation was the absence of an evaluation of the bIMGA’s precise position in their investigation. In addition, the study sample comprised only individuals who had never undergone knee surgery or exhibited any anatomical deformities. This potentially restricts the applicability of these findings, indicating that the utility of this anatomical feature may be limited to a subset of patients, according to the authors’ study results.

Based on an examination of four cadaveric specimens, Zaffagnini et al. provided a detailed description of the neurovascular anatomy [7]. Our own research indicates that the efficacy and usefulness of these anatomical landmarks remain questionable. Our study revealed a high degree of variability in the presence of the vessel, which may be attributable to inherent anatomical variations or identification difficulties. When possible, blunt palpation of the pes anserinus can provide insightful information. In certain patient populations, this technique’s efficacy may be significantly compromised. For instance, in patients with an abundance of subcutaneous adipose tissue, palpation can be especially difficult, thereby impeding accurate identification and harvesting procedure. Moreover, an analysis of a prospective series of MRI scans reveals that women tend to have a greater accumulation of fat superficial to the insertion of their hamstring tendons than males [21]. This anatomical distinction appears to be independent of Body Mass Index and may present additional complications during hamstring tendon harvesting, possibly influencing the visibility and accessibility of the insertion site.

Shahid and colleagues examined 20 cadaveric knees in detail, concentrating on the branching and vascular anatomy of the geniculate arteries [22]. In this analysis, bIMGA was consistent across all knees and the authors emphasize the importance of incorporating these extensive variations into standard surgical procedures. This consideration is crucial as it may reduce local morbidity rates. This leads us to believe that the incidence of bIMGA can be higher than our results show, but its anatomical variability is high. This can be seen as a clinical example in Figure 1, where it can be clearly perceived that on the left knee, the vascular structure has a different orientation.

The inconsistency of bIMGA as a reliable anatomical reference during graft harvesting procedures was observed in our study. This can be explained and highlighted by a number of factors. Initially, the bIMGA’s small caliber vessel size and patient-specific anatomical characteristics make it easy to overlook during surgical procedures. The absence of detection during our surgical procedures does not necessarily indicate that the vessel does not exist. Second, inherent vascular variability poses a difficult obstacle and directly contributes to the third possible explanation, which is the difficulty of precisely locating a small vascular structure via a less invasive surgical approach in a manner that can be replicated and standardized. This observation necessitates additional research to refine existing methods or identify more reliable anatomical landmarks that could improve the efficacy and predictability of graft harvesting techniques.

Obviously, the utility of prominent anatomical landmarks for various interventions and comprehensive approaches cannot be contested or changed, as they serve as navigational aids for surgeons during the procedure [8]. Our data analysis indicates a statistically significant correlation between bIMGA’s presence and levels of activity. We hypothesize that this association may be due to demographic factors such as weight and subcutaneous tissue volume, both of which may interfere with the accurate identification of bIMGA. In the analytical phase of the study, the chi-square test was employed to evaluate the association between categorical variables—specifically, the data related to distinct levels of activity. By employing this method, it was possible to determine if there was a significant difference in the distribution of observed frequencies compared to expected frequencies under the assumption of independence.

Despite the fact that the current study provides important insights into the interaction between age groups, gender, activity levels, and the detection of the bIMGA during ACLR, it is important to acknowledge our limitations. The reduced sample size, which is common in single-center research, is a restriction that may have reduced statistical power and the capacity to identify significant changes. In the design of this study, no power analysis or specific sample size calculation was conducted. The decision to omit these analyses was made in consideration of the exploratory nature of the investigation and the limited existing literature on the bIMGA in ACL reconstructions. The sample size of 213 patients was determined based on the clinical volume over a two-year period, ensuring a diverse and representative cohort for the evaluation of bIMGA. While this approach may limit the generalizability of the statistical findings, it enabled in-depth and nuanced insights into the state-of-the-art of this specific anatomical feature.

Furthermore, the study was restricted to a specific patient cohort, which may not be indicative of the broader population. Additional multicenter research with larger sample sizes and more diverse patient populations is required to validate and expand the current study’s findings.

Our study provides new insights into the challenges of identifying bIMGA in ACL graft harvesting, emphasizing the significant variability in its presence and its correlation with patient activity levels. Derivatively, we have underscored the importance of individualized approaches and meticulous preoperative evaluations highlighting the thoroughly described importance of personalized evaluations prior to surgery [23,24,25]. Our findings also illuminate the need for refining surgical methods and identifying more reliable anatomical references. Despite certain limitations, our research contributes valuable perspectives to the evolving body of knowledge on this specific anatomical feature in ACL reconstructions. Moreover, in this realm of ACL reconstructions, advancements are ceaselessly shaped by nascent research findings and technological innovations. Our scholarly contributions to this continually developing discourse underscore the imperative for sustained academic inquiry and methodological evolution.

A better understanding and appreciation of these anatomical variances can help to optimize surgical methods, lowering the chance of unfavorable outcomes. Our findings highlight the necessity of individualized surgical and therapeutic approaches based on thorough preoperative evaluations.

## 5. Conclusions

Based on our examination of the studied sample, bIMGA demonstrated a high degree of anatomical variation, thereby posing substantial challenges in its consistent identification within the surgical field. Our analysis did not establish a significant correlation between the variability of the bIMGA and the demographic characteristics of patients. Considering these observations, we advise against the utilization of the bIMGA as a reliable anatomical landmark during ACL graft harvesting procedures due to its marked inconsistency and the potential to compromise surgical precision.

## Figures and Tables

**Table 1:** Patient demographics and characteristics.

Patient Characteristics	Total *n* = 213
Gender (*n*, %)	
Male	135 (63.4%)
Female	78 (36.6%)
BMI (mean ± sd)	
Male	26.4 ± 3.7
Female	23.7 ± 3.4
Smoking status (*n*, %)	
Current smoker	38 (17.8%)
Past smoker	25 (11.7%)
Non-smoker	150 (70.5%)
Alcohol use status (*n*, %)	
Regular use	46 (21.6%)
Occasional use	101 (47.4%)
Non-drinker	66 (31%)
Conservatory treatment post-injury (*n*, %)	
Yes	100 (47%)
No	113 (53%)

BMI—Body Mass Index; sd—standard deviation; *n*—number.

**Table 2:** Age of patients and their activity level (number of patients corresponding to each age interval—also depicted in percentage; number of patients corresponding to each activity level group).

		Male	Female
	TOTAL	135 (63.38%)	78 (36.62%)
**Age (*n*, %)**	Under 20	13 (9.62%)	19 (24.35%)
	20–30	57 (42.22%)	19 (24.35%)
	30–40	28 (20.74%)	24 (30.76%)
	40–50	7 (5.18%)	10 (12.82%)
	Over 50	7 (5.18%)	6 (7.69%)
Activity level * (*n*)	1	10	11
	2	16	19
	3	53	23
	4	50	20
	5	6	5

*n*—number. * Activity level scales from 1 to 5.

**Table 3:** The findings of our bIMGA during our harvesting approach for different age intervals, genders, and activity levels.

		Male		Female	
		bIMGA Present	bIMGA Absent	bIMGA Present	bIMGA Absent
	TOTAL	73 (54%)	62 (46%)	26 (33.3%)	52 (66.6%)
**Age (*n*)**	Under 20	9	4	12	7
	20–30	32	25	6	13
	30–40	18	10	4	20
	40–50	7	15	3	7
	Over 50	7	8	1	5
Activity level * (*n*)	1	6	4	4	7
	2	8	8	6	13
	3	29	24	7	16
	4	25	25	8	12
	5	5	1	1	4

* rated on a scale of 1–5; 1—very low, 5—very high.

**Table 4:** Statistical significance of variables and bIMGA identification.

	*t*-Test *
BMI	0.085
Age	0.062
Smoking Status	0.062
Gender	0.073
Alcohol Use	0.079

* *p*-value.

## Data Availability

The data presented in this study are available on request from the corresponding author. The data are not publicly available due to ethical considerations.
